# Supplementary material for: Establishment of a Perfusion Process with Antibody-Producing CHO Cells Using a 3D-Printed Microfluidic Spiral Separator with Web-Based Flow Control
Source: Bioengineering (Basel). 2023 May 28;10(6):656. doi: 10.3390/bioengineering10060656 (PMC10295792; doi:10.3390/bioengineering10060656)
Supplement: Supplementary file 1 [file bioengineering-10-00656-s001.zip › bioengineering-2389417-supplementary.pdf]

Supporting Information

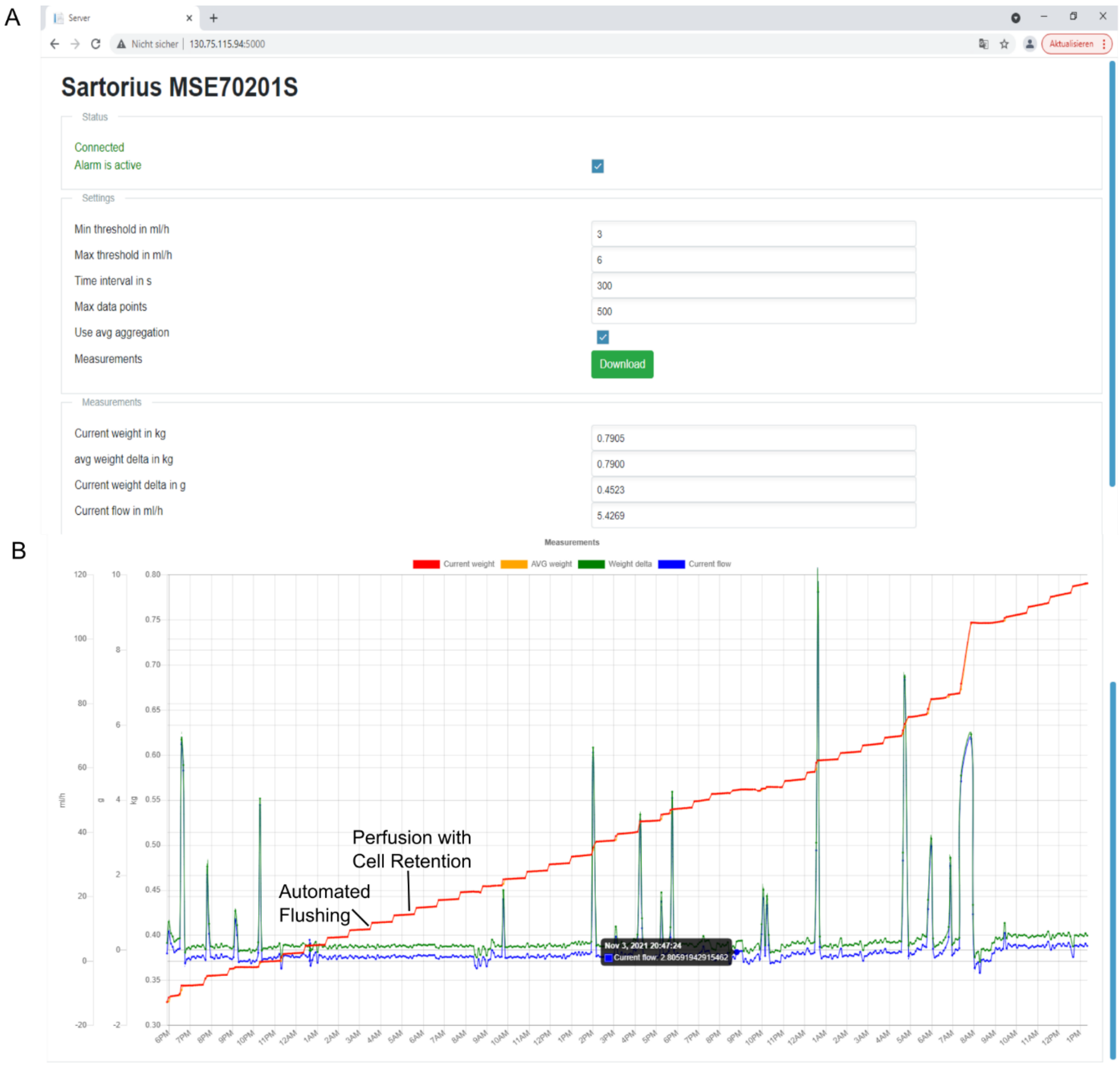

Figure S1. Illustration of the web-based flow control and monitoring. A) Interface with the possibility of settings adjustment; B) Graphical representation of the measured weight and the calculated flow rate.

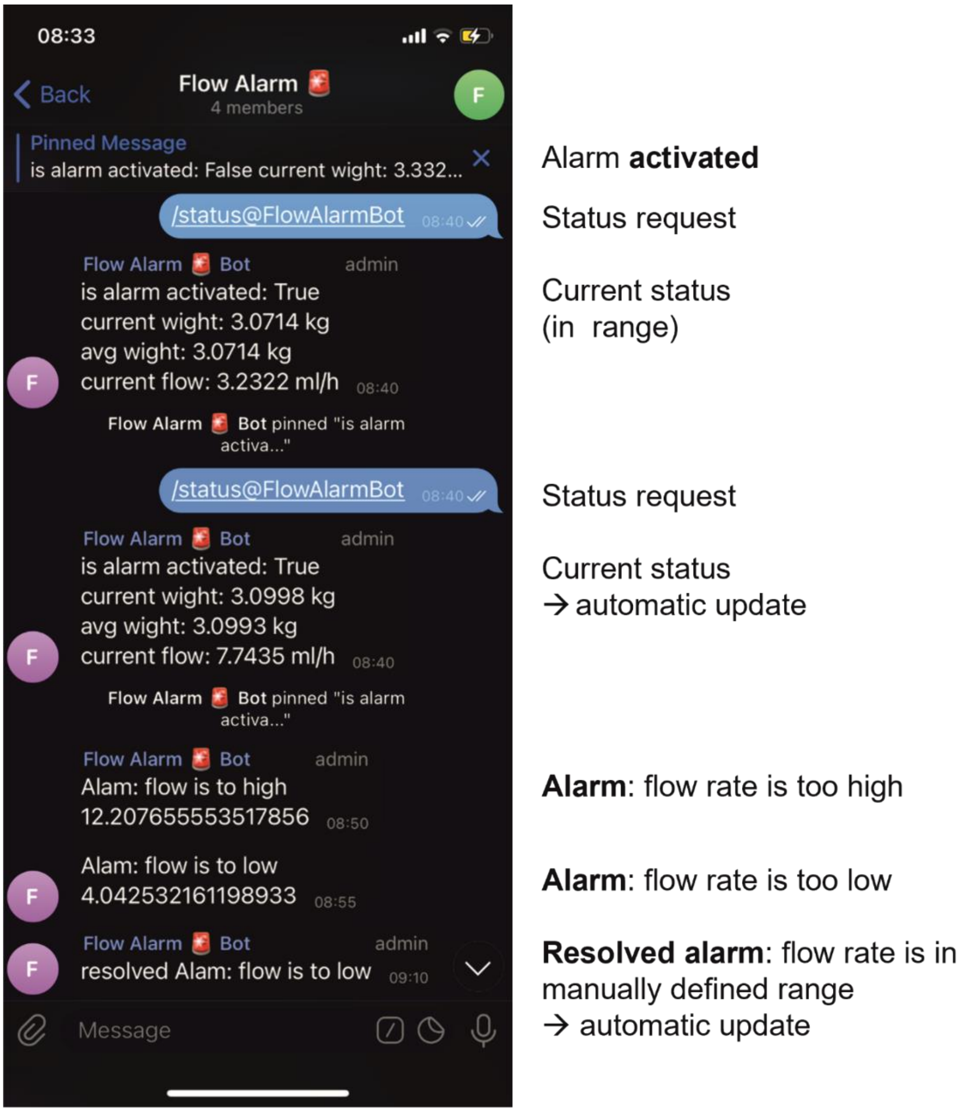

Figure S2. Display of the Telegram bot with status requests, active alarms and the resolution of alarms.

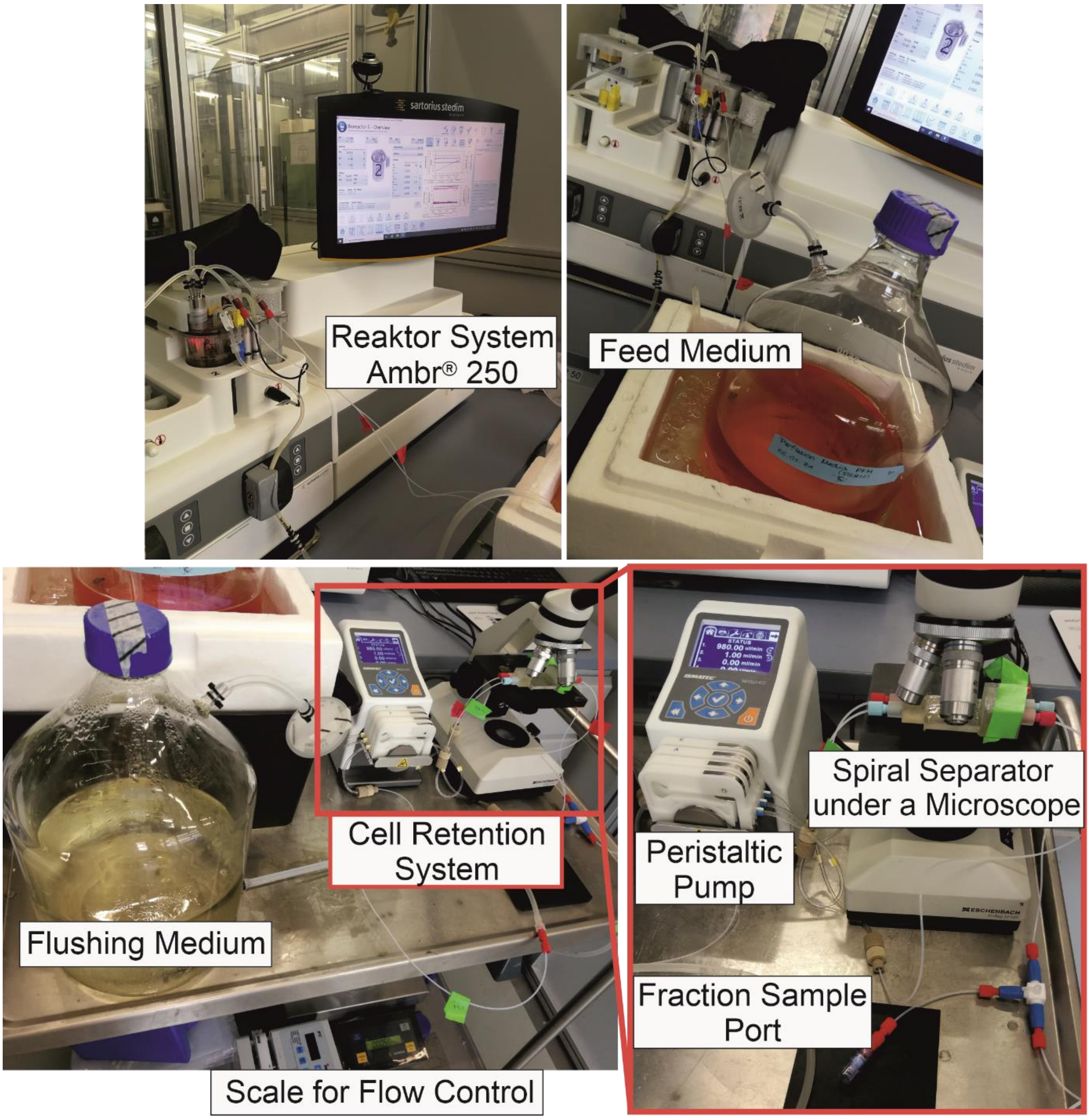

Figure S3. Photographs of the experimental setup in the laboratory.

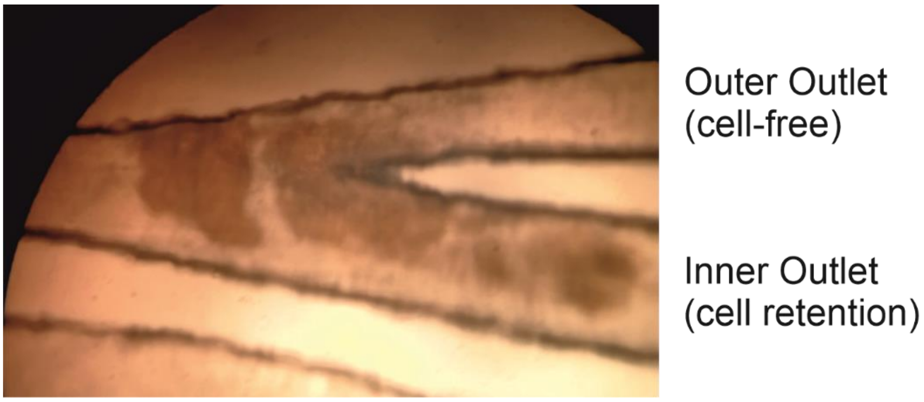

Figure S4. Microscopic image of the spiral separator channel split before flushing when clogged with cell aggregates (Cedex HiRes: Magnification 10X). Here, the automatic flushing system was implemented to continuously reduce and remove blockages at higher cell concentrations.
